# The Importance of miRNA Identification During Respiratory Viral Infections

**DOI:** 10.33696/immunology.3.101

**Published:** 2021

**Authors:** Ivan Martinez-Espinoza, Ma Del Rocio Banos-Lara, Antonieta Guerrero-Plata

**Affiliations:** 1 Department of Pathobiological Sciences, Louisiana State University, Baton Rouge, LA, USA; 2 Universidad Popular Autonoma del Estado de Puebla, Mexico

**Keywords:** Respiratory viruses, miRNAs, Respiratory tract, Non-coding RNAs, HMPV, RSV, HRSV

## Abstract

The expression of small non-coding RNA MicroRNAs (miRNAs) during respiratory viral infections is of critical importance as they are implicated in the viral replication, immune responses and severity of disease pathogenesis. Respiratory viral infections have an extensive impact on human health across the globe. For that is essential to understand the factors that regulate the host response against infections. The differential miRNA pattern induced by respiratory viruses has been reported, including include influenza A virus (IAV), human respiratory syncytial virus (HRSV), human metapneumovirus (HMPV), adenovirus (AdV), and more recently, severe acute respiratory syndrome coronavirus-2 (SARS-CoV-2) infection. In this commentary, we highlight the importance of miRNAs identification and the contribution of these molecules in the modulation of the immune response through the upregulation and downregulation of miRNAs expression in different immune and non-immune cells.

## Introduction

MicroRNA (miRNAs) constitute a large family of highly conserved ~21-nucleotide-long small noncoding RNAs. In mammals it is calculated to control ~50% of protein-coding genes by regulation at the post-transcriptional level affecting mRNA degradation [1–3]. This interaction, miRNA-mRNA commonly increases degradation of mRNA by pairing to complementary sequences at the 3Úntranslated Region (3´-UTR) and other important gene sequences repressing translation into protein or promoting mRNA deadenylation [4–6]. Therefore, miRNAs play a role in modulating a variety of biological processes such as development, immune system response and cell death [7–10]. They represent a potential explanation for viral replication and proliferation, host antiviral response, viral pathogenesis in the modulation of inflammatory responses, and their role in the refinement of the innate immune defense against different viruses, diagnostics, and treatment [7,8,11–14].

## Differential Induction of miRNAs by HMPV and HRSV in Dendritic Cells

**Figure 1:**
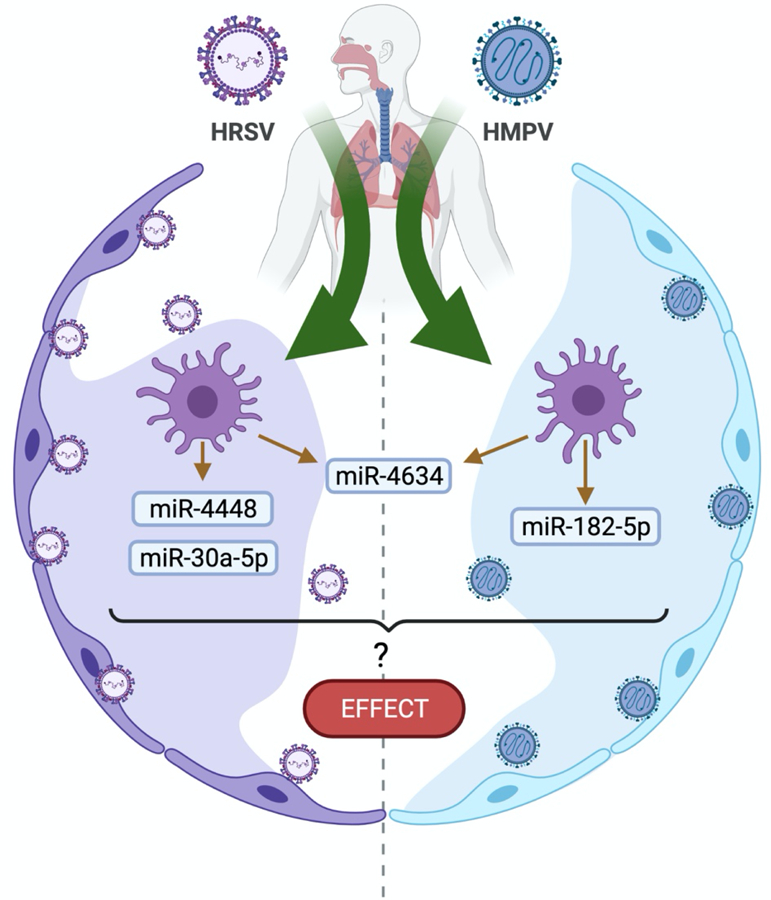
Upregulation of miRNA expression by HMPV and HRSV infection in monocyte derive dendritic cells (MoDCs). [15]. Identification of the miRNAs was conducted by the side-by-side analysis of miRNAs expressed in moDCs infected with HMPV or HRSV by nextgeneration sequencing followed by validation by qPCR. MoDCs infected with HRSV induced miR4448 and miR-30a-5p, whereas HMPV upregulated the expression of miR-182–5p. Both viruses induced significant expression of miR-4634. Future investigation is necessary to determine the regulatory effect of these miRNAs in the infected cells [15]. Figure created using BioRender.com.

During viral infections some miRNAs are upregulated or downregulated in a large variety of immune cells such as dendritic cells, granulocytes, monocytes, macrophages, and natural killer cells [11,12]. We have previously reported that human metapneumovirus (HMPV) induces a distinct pattern of miRNAs than that of human respiratory syncytial virus (HRSV) in human monocyte-derived dendritic cells (moDCs) [15]. HMPV and HRSV are single-stranded RNA pneumoviruses that cause upper and lower respiratory tract infections (LRTI) in children worldwide [16–21]. We observed that when HMPV infects human moDCs, it induced the expression of hsa-miR-182–5p and hsa-miR-4634 predominantly, while HRSV infection induced a different pattern of miRNAs that included hsa-miR-448, hsa-miR-30a-5p and hsa-miR-4634. Overall, the predominant miRNA induced by both viruses was hsa-miR-4634 (Figure 1) [15]. These findings are relevant since viral infections induce a specific miRNA pattern in different target cells and can differentially modulate the immune response. Future research is warranted to investigate the role of these miRNAs in HMPV and HRSV infection and how the expression of these miRNAs would alter the host immunity as cellular miRNAs induced by other respiratory viruses have been reported to modulate the immune response.

## miRNAs and Their Role in Respiratory Viral Infection Immunity

Respiratory viral infections have an extensive impact on human health. Some respiratory viruses have been reported to induce miRNAs implicated in viral replication and immune response modulation[22]. Some examples include influenza A virus (IAV) [7,7,23–28], human respiratory syncytial virus (HRSV) [15,29–34], human metapneumovirus (HMPV) [15,21], adenovirus (ADV) [35], and due to the global health crisis miRNAs have been studied recently in SARS-CoV-2 infection[36].

IAV induces several miRNAs that alter the immune response, including the miR-136 [37], and miR-132–3p, which targets IRF1, inhibiting IFN response and promoting IAV replication [25]. IAV also induces miR-29c expression, which correlates with decreased NF-κB activity and expression of TNF-α, IFN-β, IL-6, IL-1b and IL-8 via the deubiquitinating enzyme A20 [26]. Similarly, IAV H3N2, induces miR-146 a-5p expression and regulates TRAF6 in in human nasal epithelial cells [27]. On the other hand, IAV H5N1 diminishes levels of miR-21–3p in infected patients and A549 cells. miR-21–3p, downregulates IFN response (IFN-β, IFN-α, PKR, MxA, and OAS) by targeting the regulating fibroblast growth factor 2 (FGF2) [28]. It has also been shown that IAV H5N1 downregulates the miRNA family (30-a, -b, -c, -d, and -e) in cell lines. The expression of SOCS1 and SOCS2 is downregulated by IAV H5N1, abrogating the IFN/JACK/STAT pathway’s inhibition effect. miRNA 30 family also inhibits the expression of the E3 ubiquitin ligase NEDD4, which negatively regulates IFITM3 [38].

HRSV infection is the main cause of hospitalization due to respiratory disease in infants and young children [31,32,39]. It is one of the respiratory viruses most extensively studied for its induction of miRNAs. Data from blood samples identified 168 miRNAs in patients with severe HRSV-associated pediatric pneumonia and 131 miRNAs in patients with mild HRSV-associated pediatric pneumonia, which might be involved in the NF-κB and MAPK signaling pathways, both essential components of the immune response [29]. When blood samples from children with severe HRSV-caused pneumonia were analyzed, four miRNAs (miR-1271–5p, miR-10a-3p, miR-125b-5p, and miR-30b-3p) were found upregulated [29]. However, when blood samples from patients hospitalized with HRSV infection were analyzed, a different pattern of overexpressed miRNA was observed (miR-106b-5p, miR-20b-5p, and miR-342–3p) [33], suggesting that the host response shapes the induction of the miRNA pattern.

The expression of miRNAs induced by HRSV has also been studied in primary cells and cell lines. Our reported study focused mainly on peripheral blood monocyte-derived dendritic cells (MoDC), where we observed HRSV infection upregulates the expression of miR-4448, miR-30a-5p, and miR-4634 [15]. Thornburg et al. has also reported the expression of miR-Let-7b by HRSV in moDCs [31]. Work on A549 cells, a human lung-derived epithelial cell line, has shown the upregulation of miR-let-7f, miR-24, miR-337–3p, miR-26b, and miR-520a-5p [32]. Additionally, in NHBE cells (primary human bronchial/tracheal epithelial cells), HRSV increased levels of miR-let-7c, miR-let-7i, and miR-30b [31]. These observations indicate that the miRNA pattern induced by the virus depends on the type of cell infected.

Downregulation of miRNAs due to HRSV infection also has been reported. In A549 cells, HRSV repressed miR-198 and miR-595 [32]; while in blood samples from patients with HRSV infection, a different pattern of miRNAs was downregulated (miR-320e, miR-320d, miR-877–5p, miR-122–5p, and miR-92b-5p) [33]. In nasal samples from infected patients, however, miR-140–5p was found reduced [34].

Although the target genes affected by the HRSV-induced miRNAs and the mechanisms of the alterations in the immune response are not fully understood, some partial mechanisms and target genes have been identified. miR-30b appears to be induced through the NF-κB pathway [40], while miR-Let-7b appears to be increased by IFN-β [31]. In A549 cells, miR-let-7f was identified as a regulator of CCL7 and SOCS3, genes involved in cytokine response against viruses, in a mechanism involving the HRSV attachment protein [32]. The upregulated miRNAs (miR-1271–5p, miR-10a-3p, miR-125b-5p, and miR-30b-3p) found in blood samples from infected patients have predicted target genes involved in the NF-kB and the MAPK signaling pathway, and T cell receptor signaling proteins (TNFRSF19, HMOX1, TLR4, LCK, and ZAP70) [29]. Likewise, overexpression of miR-106b-5p, miR-20b-5p, miR-342–3p, miR-320e, miR-320d, miR −877–5p, miR-122–5p, and miR-92b-5p may be involved in inflammatory and immune responses by likely targeting the activation pathways of TGF-β, insulin, and T and B cell receptors [33]. On the other hand, the reduced expression of miR-140–5p in nasal and blood samples of patients with HRSV infection led to the downregulation of TNF-α, and IL-1β, −6 and −8, mediated by IFN-α (in BEAS-2B cells). In bronchial epithelial cells, the downregulation of miR-34b/c-5p resulted in increased expression of Muc5ac, probably through of c-Jun activation pathway [39].

Human metapneumovirus (HMPV) belongs to the *Pneumoviridae* family and is the first human member of the *Metapneumovirus* genus [18,20]. This virus presents similar pathology and clinical symptoms as the ones of HRSV. In both cases coughing, bronchitis, bronchiolitis and respiratory manifestations related to the absence of airflow are seen [41,42]. HMPV affects all age groups worldwide, with a risk of sever disease found in immunocompetent patients, children and elderly [43]. HMPV infection alters the expression of multiple cellular miRNAs in several cells [21]. We reported that HMPV upregulated significantly the expression of miR-182–5p, and miR-4634 in moDCs, where we highlighted the upregulation of miR-182–5p [15], which might contribute to the imbalance of Th17 cells [44] as shown in unrelated models where miR-182–5p triggers the secretion of cytokines related to a Th2-like profile, leading to high recruitment of neutrophils in the lung and secretion of cytokines such as IL-13 and IL-5 [16], and protects against different intracellular pathogens, increasing the expression of interferons and other proinflammatory cytokines [45,46]. In A549 cells, the miRNAs expression pattern is different. HMPV induced the expression of miR-Let-7f, miR-192, miR-374a, and miR-452, which are upregulated by HMPV, where miR-Let-7f appears to favors the replication of HMPV [21] through an unknown mechanism.

Like HMPV and HRSV, another cause of pneumonia in infants and children is the human adenovirus (HAdV) infection. The expression profile of whole blood samples from healthy and infected patients with HAdV, indicated the expression of 3 upregulated miRNAs (miR-127–3p, miR-493–5p, and miR-409–3p). Such miRNAs were found to target PSMB5, ITGA6, MYCBP2, TCF7L2, UBE2V2, HIPK1, UBE2D2, and KANSL1genes, mostly involved in cellular processes, and MAPK and Ras signaling [35].

In search of therapeutic targets against the severe acute respiratory syndrome coronavirus-2 (SARS-CoV-2) infection, miRNAs induced by this virus have also been investigated [47]. Lu et al. studied the miRNAs differentially expressed in blood samples from patients with COVID-19, and they found that miR-16–2-3p, miR-6501–5p, and miR-618, miR183–5p, miR-627–5p, and miR-144–3p were upregulated in COVID-19 positive patients compared to healthy individuals [47]. Analyses *in silico* on the SARS-CoV-2 genome sequence have revealed matching of viral genes with some elements involved in the immune response. Specifically, the protein S exhibits miRNA-like sequences that match with IFNA7 and IRF1; but, if these interactions occur in the infection, and their biological implications remained to be explored [48]. Likewise, Aydemir et al. found 40 SARS-CoV-2 miRNAs, which target genes involved in the immune response through NF-κB, JAK/STAT and TGF-β signaling pathways. However, the *in vivo* relevance of these molecules is so far unknown [49].

## Conclusions and Perspectives

The upregulation or downregulation of miRNAs during respiratory viral infections can act differently in the host by altering multiple processes, including the immune response. The presence of specific miRNAs can modulate the immune response by inhibiting or activating genes involved in the expression of inflammatory cytokines, IFN response, cellular receptors, and other immune processes. On the other hand, miRNA expression can affect viral replication by increasing or decreasing the viral gene expression. Strikingly, miRNAs can have a paracrine effect on other non-infected cells to activate the immune response. Future research is warranted to investigate the profiles of miRNA expression induced by viral infections in the respiratory tract in the different immune and nonimmune cells to understand more on how miRNAs are involved in viral pathogenesis. Furthermore, it is observed that viruses can induce different miRNA profiles but exert a similar effect on the host immune response. That phenomenon may result from having different miRNAs targeting the expression of distinct proteins, but regulating similar immune pathways, e.g., different miRNAs targeting diverse cellular receptors that trigger an IFN response. That scenario could represent a viral survival strategy allowing the virus to replicate more efficiently. Therefore, further research on the functional validation of the molecules targeted by the diverse miRNAs is essential to fully understand the regulatory mechanisms induced by viral infections.

Although the use of miRNAs as therapeutic agents remains largely under development, there are promising data using specific miRNA antagonists (antagomirs) to treat viral infections. That is indicated by the clinical trials testing the oligonucleotide Miravirsen (SPC3649) targeting miR-122 for the treatment of hepatitis C infection [50,51]. However, more information on the identification of miRNAs and their targets, as well as on the characterization of the underlying molecular mechanisms of miRNA-mRNA regulation during viral infections is critical to design improved antiviral therapies for respiratory viral infections.

## Figures and Tables

**Table 1:** Differential induction of miRNA by human respiratory viral infections.

VIRUS	miRNA	EFFECT	CELL	REFS.
IAV H1N1	↑miR-132-3p	Suppresses type I IFN response and promotes IAV replication	A549 cells	[25]
	↑miR- 29c	Decreases NFκB, TNF-α, IFN-β, IL-6, IL-1β and IL-8		[26]
IAV H3N2	↑mir-146 a-5p	Regulates TRAF6	Human nasal epithelial cells	[27]
IAV H5N1	↑miR-136	Acts as an immune agonist of RIG-I, thus leads to IL-6 and IFN-β accumulation	A549 cells	[37]
	↑miR-21-3p	Decreases the levels of IFN-β, IFN-α, PKR, MxA, and OAS		[28]
	↓miR-30a,-b,-c,-d,-e.	Modulates SOCS1, SOCS3, and NEDD4 to impair the host antiviral response		[38]
HRSV	↑miR-1271-5p	Undefined	Whole blood samples	[29]
	↑miR-10a-3p			
	↑miR-125b-5p			
	↑miR-30b-3p			
	↑miR-106b-5p	Undefined	Peripheral blood	[33]
	↑miR-20b-5p			
	↑miR-342-3p			
	↓miR-320e			
	↓miR-320d			
	↓miR-877-5p			
	↓miR-122-5p			
	↓miR-92b-5p			
	↓miR-34b/c-5p	Induces Muc5ac expression	Human bronchial epithelial cells	[39]
	↑miR-Let-7b	Regulates IFN-β response	MoDCs	[31]
	↑miR-4448	Undefined	MoDCs	[15]
	↑miR-30a-5p			
	↑miR-4634			
	↑miR-let-7f	Undefined	A549 cells	[32]
	↑miR-24			
	↑miR-337-3p			
	↑miR-26b			
	↑miR-520a-5p			
	↑miR-let-7c	Undefined	Normal human bronchial epithelial cells	[31]
	↑miR-let-7i	Regulates IFN-β response		
	↑miR-30b	Undefined		
	↓miR-198	Undefined	A549 cells	[32]
	↓miR-595			
HMPV	↑miR-182-5p	Undefined	MoDCs	[15]	
	↑miR-4634				
	↑miR-Let-7f	Regulates viral replication	A549 cells	[21]	
	↓miR-192	Undefined			
	↓miR-374a				
	↑miR-452				
HAdV	↑miR-127-3p	Undefined	Whole blood samples	[35]	
	↑miR-493-5p				
	↑miR-409-3p				
SARS-CoV-2	↑miR-16-2-3p	Undefined	Whole blood samples	[47]	
	↑miR-6501-5p				
	↑miR-618				
	↓miR183-5p				
	↓miR-627-5p				
	↓miR144-3p				
